# Influence of Auditory Integrative Training on Casein Kinase 2 and Its Impact on Behavioral and Social Interaction in Children with Autism Spectrum Disorder

**DOI:** 10.3390/cimb45050274

**Published:** 2023-05-15

**Authors:** Laila Al-Ayadhi, Ramesa Shafi Bhat, Farah Ali Alghamdi, Abdulmalik S. Alhadlaq, Afaf El-Ansary

**Affiliations:** 1 Department of Physiology, Faculty of Medicine, King Saud University, Riyadh 11495, Saudi Arabia; lyayadhi@ksu.edu.sa; 2 Autism Research and Treatment Center, Riyadh 12713, Saudi Arabia; 3 Biochemistry Department, College of Science, King Saud University, Riyadh 11495, Saudi Arabia; rbhat@ksu.edu.sa; 4 College of Medicine, Dar Al Uloom University, Riyadh 13314, Saudi Arabia; 2186861@du.edu.sa; 5 College of Medicine, King Saud University, Riyadh 11495, Saudi Arabia; aahadlaq@gmail.com; 6 Autism Center, Lotus Holistic Medical Center, Abu Dhabi 110281, United Arab Emirates

**Keywords:** autism spectrum disorders, casein kinase-2, auditory integration therapy, childhood autism rating scale, social responsiveness scale, short sensory profile

## Abstract

Considerable disturbances in post-translational protein phosphorylation have recently been discovered in multiple neurological disorders. Casein kinase-2 (CK2) is a tetrameric Ser/Thr protein kinase that phosphorylates a large number of substrates and contributes in several cellular physiological and pathological processes. CK2 is highly expressed in the mammalian brain and catalyzes the phosphorylation of a large number of substrates that are crucial in neuronal or glial homeostasis and inflammatory signaling processes across synapses. In this study, we investigated the impact of auditory integration therapy (AIT) for the treatment of sensory processing abnormalities in autism on plasma CK2 levels. A total of 25 ASD children, aged between 5 and 12 years, were enrolled and participated in the present research study. AIT was performed for two weeks, for a period of 30 min, twice a day, with a 3 h interval between sessions. Before and after AIT, the Childhood Autism Rating Scale (CARS), Social Responsiveness Scale (SRS), and Short Sensory Profile (SSP) scores were calculated, and plasma CK2 levels were assayed using an ELISA test. The CARS and SRS indices of autism severity improved as a result of AIT, which could be related to the decreased level of plasma CK2. However, the mean value of the SSP scores was not significantly increased after AIT. The relationship between CK2 downregulation and glutamate excitotoxicity, neuro-inflammation, and leaky gut, as etiological mechanisms in ASD, was proposed and discussed. Further research, conducted on a larger scale and with a longer study duration, are required to assess whether the cognitive improvement in ASD children after AIT is related to the downregulation of CK2.

## 1. Introduction

Autism spectrum disorder (ASD) is a neurodevelopmental disorder which is characterized by impaired social interaction and communication, stereotypic and restricted behaviors, and abnormal sensory reactivity. Within the last several decades, the incidence rate of ASD has increased dramatically, and cases of ASD have been reported at a rate of 0.6–0.8% in infants, and 1.0% in school-age children and young adults [1,2]. Under this circumstance, it is urgent to understand the mechanisms involved in the development of ASD to enable the early detection of biomarkers that could help improve timely diagnosis, with a significant influence on lifelong prognosis [3]. 

Most proteins in mammalian cells are phosphorylated as a dynamic post translational modification that can control protein folding, interactions, localization, and stability [4,5,6]. Phosphorylation adds two negative charges to the protein at physiological pH, which will modify the electrostatic milieu and can change the strength of protein–protein interactions [4,6]. Although the estimated stability of most protein complexes is not altered by phosphorylation, about one-third of these complexes is expected to be significantly stabilized or destabilized by phosphorylation.

Casein kinase-2 (CK2) is a tetrameric Ser/Thr protein kinase that phosphorylates a large number of substrates and contributes to numerous cellular physiological and pathological processes, such as proliferation, survival, apoptosis, angiogenesis, endoplasmic reticulum stress response, DNA damage and repair, carbohydrate metabolism, and most importantly, brain development [7]. CK2 is regularly expressed in the periphery, as well as in the brain [8]. However, there is currently insufficient information regarding the role of CK2 in brain development. Lettieri et al. [8] reported that the loss of CK2 α’ and β subunits severely interrupts GN11 neuronal cell line migration, as well affects cell adhesion, through the activation of diverse signaling pathways, thus providing the first proof of CK2 importance in neuron migration. This may be related to the observation that mutations in genes encoding for CK2 subunits have been identified in patients clinically presenting with NDDs, supporting the idea that CK2 is certainly essential for appropriate neuronal migration during brain development.

CK2 has been identified in the plasma membrane, as well as in the nucleus and cytoplasm of neurons [9], more specifically at the postsynaptic density in rat hippocampus and cortical preparations [10]. CK2 activity is enhanced in synaptosomes [11], and a plethora of CK2 substrates discovered in vitro or in vivo convincingly link CK2 to synaptic activity modulation [12]. CK2 regulates the homeostasis of neurotransmitter receptors such as ion channel receptors and G-proteins coupled receptors (GPCRs) [13,14]. The NMDA glutamate receptor, a cation channel for Ca^2+^, Na^+^, and K^+^, plays important roles in synaptic plasticity, memory, and learning.

Abnormal neuronal migration in individuals with autism, as well as in rodent models, has been detected in most brain regions relevant to autistic behaviors [15]. Among the most common recorded abnormal migration in ASD are the migration of GABAergic interneuron and glutamatergic neuron cell types [16,17]. This can be related to the well-documented reduced GABAergic inhibitory tone which occurs in autistic brains [18], resulting in the altered excitation/inhibition (E/I) balance of ASD-relevant brain circuits [18,19].

In relation to ASD, it is interesting to note that CK2 interacts with the autism susceptibility candidate 2 (AUTS2) gene as an emerged crucial gene associated with a wide range of neurodevelopmental disorders, among which is ASD [20,21]. Okur et al. [22] postulate that the mutations that alter CK2 function and the phosphorylation of CK2 targets lead to deleterious effects on brain development and function. It has been proposed that CK2 is a cause of syndromic developmental delay, and perhaps autism spectrum disorder [23].

Furthermore, CK2 has been proposed as the primary α-synuclein Ser129 kinase in the brain. This could support the link between CK2 levels in ASD individuals and the severity of the disease. Alpha-synuclein phosphorylation was first proposed as a factor in protein toxicity and aggregation formation. Surprisingly, in subjects with neurological diseases, almost 90% of α-synuclein Ser129 sites are phosphorylated, compared to only 4% or less in subjects without these diseases. CK2 inhibition appears to be advantageous in a variety of neurological conditions, including ASD, according to various studies [7,24]. Increased plasma levels of α-synuclein have recently been reported in people with ASD and have been linked to the development and severity of the illness [25,26,27,28].

Intestinal permeability reveals the sum of the functionally discrete tight junction pore and the leakage pathways. The tight junction pore pathway is a high capacity, size- and charge-selective passageway whose permeability is mostly controlled by a subset of claudin family proteins [29,30]. Interestingly, it was found that the inhibition of CK2 blocks claudin-2 channel function through the prevention of IL-13-induced claudin-2 upregulation, increasing gut permeability in vivo [31]. Moreover, Dörfel et al. [32] reported that CK2-dependent occludin phosphorylation impaired its binding with zonulin-2 protein, greatly affecting tight junction integrity, and thus increasing intestinal permeability. 

Although the most effective approach to intervention is not clear, the scenario of ASD patients can be improved with early intervention [33]. Auditory integrative training (AIT) was developed to enhance aberrant sound sensitivity, especially for behavioral disorders such as ASD [34]. AIT may be helpful as an intervention technique for people with ASD, according to Rotschafer et al. [35], due to the fact that anomalous sensitivity or insensitivity to specific sound wave frequencies, regardless of overall hearing capacity, is related to some form of behavior and learning difficulties.

Considering the crucial role of CK2 in the development of the brain [14], it is of great interest to study the role of CK2 in the etiology of autism. Therefore, the purpose of this study was to investigate potential AIT effects on plasma CK2 levels, as well as the relationship between these levels and the improvement of social and cognitive impairment in AIT-trained individuals.

## 2. Materials and Methods

**Scheme 1:**
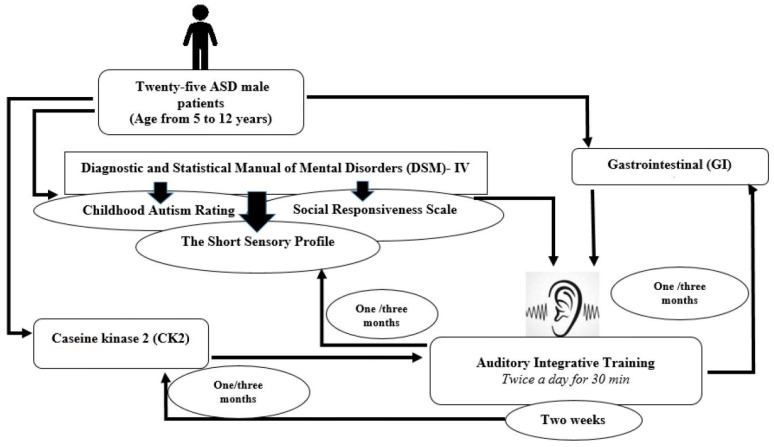
A schematic design of the study.

The Autism Research and Treatment Center at King Khalid University Hospital in Riyadh, King Saud University, and the Kingdom of Saudi Arabia were the sources of participants for this study. The study included 25 male ASD patients, with ages ranging from 5 to 12 years. Utilizing the Diagnostic and Statistical Manual of Mental Disorders (DSM-IV), all individuals were tested and assessed. The CARS, SRS, and SSP scores were computed prior to and following intervention (i.e., immediately after, one month, and three months after AIT for each child). AIT sessions were conducted twice daily for two weeks, with each session lasting 30 min, and 3 h breaks in between sessions. The study excluded children with a history of seizures. The schematic design of the study is summarized in Scheme 1.

Written consent was obtained from the parents of each patient, according to the guidelines of the Ethics Committee of the King Saud University, King Khalid University Hospital. Children were not permitted to start new treatments, or terminate existing treatments, including the use of prescription drugs and dietary supplements, during the AIT intervention period. The Institutional Review Board of the College of Medicine at King Saud University granted ethical approval for the study.

### 2.1. Childhood Autism Rating Scale

The CARS score was evaluated as a measurement of the severity of autism. The child is assessed by using a scale of 1 to 4 for each of 15 characteristics or symptoms, including verbal communication; listening response; fear or nervousness; imitation; body use; object use; ability to relate to people; emotional response; nonverbal communication; activity level; level and reliability of intellectual response; adaptation to changes; visual response; taste, smell, and touch responses; and general impressions. The presence of autism is strongly suggested by a total score of at least 30. While children with scores between 37 and 60 have severe autism, those between 30 and 36 have mild-to-moderate autism [36].

### 2.2. Social Responsiveness Scale

The SRS is an authorized test of social behavior, stereotypical traits, and communication in autism [36]. It is used as a diagnostic instrument, differentiating clinically presenting ASD children from others with different levels of social interaction impairment, but who exhibit non-ASD psychiatric disorders. It consists of 5 subscales: (1) social awareness, (2) social cognition, (3) social communication, (4) social motivation, and (5) autistic mannerisms. Total SRS scores range from 0 to 195, following or evaluating the significant social impairment as observed in individuals with ASD. A score between 60 and 75 is in the mild-to-moderate range of social impairment, while a score of 76 or higher is considered severe and is strongly associated with a clinical diagnosis of ASD [37]. 

### 2.3. The Short Sensory Profile

The 38-item SSP questionnaire, which is intended for kids aged 3 to 14, offers brief details regarding the sensory-processing abilities of autistic kids [38]. Each SSP item is scored on a 5-point Likert scale. Domain scores for the areas of movement sensitivity, wanting sensation, auditory filtering, low energy levels, and visual/auditory sensitivity were measured. The categories of typical performance, probable variation from usual performance, and definitely different from typical performance were used to evaluate domain scores and overall sensory responses. Scores between 143 and 152 indicate mild-to-moderate performance (probably different from typical performance), scores between 153 and 190 show typical performance, and scores below 142 represent severely different performance (clear deviation from typical performance). Numerous studies have employed the SSP [39]. The Auditory Integration Training AIT was conducted in accordance with a published technique previously used by our team [40].

Participants were initially examined by a medical doctor to confirm that there was no wax and/or fluid in their ears. They then participated in 20–30-min hearing sessions for a 15- to 20-day period, with a break of 1 or 2 days after 5 therapy days. The child listened to recorded music during these sessions. The AIT sound amplifier attenuated low and high frequencies from the CDs at random before sending the changed music to the listener through headphones. Depending on the person’s comfort level, the volume during the AIT sessions was set at much lower intensities and did not go above 80 dBA (low scale). The volume of the music was generally considered to be safe.

### 2.4. Measurement of Plasma Casein Kinase-2 (CK2)

Fasting blood samples from each child were collected in test tubes containing EDTA, the samples were immediately centrifuged at 3000 rpm, and plasma was collected and stored at −80 °C until analysis. All samples were assayed in duplicate and in a double-blind manner. CK2 concentrations were measured in the plasma of autistic subjects using a commercially available sandwich ELISA kit, a product of Cusabio Biotech Co. Ltd., Wuhan, China. Plasma CK2 levels were measured before AIT and again immediately after, one month after, and three months after AIT, according to the manufacture instructions. To improve accuracy, all samples in the current investigation were tested in two independent trials as duplicates to confirm repeatability and detect inter-assay variances in the results (*p* < 0.05). No significant interference or cross-reactivity was identified.

### 2.5. Statistical Analysis

The Statistical Package for the Social Sciences (SPSS 21.0 for Windows; SPSS, Chicago, IL, USA) was used to analyze the data. Mean SD was used to express results. Using repeated-measure analysis of variance, significant measured changes in the parameters were evaluated. Significant differences were also evaluated using Bonferroni multiple comparison tests.

The receiver operating characteristic (ROC) analysis approach was used to evaluate the prognostic and predictive value of CK2 after AIT treatment in relation to CARS, SRS, and SP as three measures of ASD severity. ROC is common statistical tool used for evaluating the diagnostic validity of biomarkers commonly used in clinical psychology. To accomplish this evaluation, a cut-off point must be set. The area under the ROC curve (AUROC) can be used to estimate how well a diagnostic variable is performing. A random prediction would have an AUROC of 0.5, while the perfect test would have an AUROC of 1.

## 3. Results and Discussion

**Figure 1:**
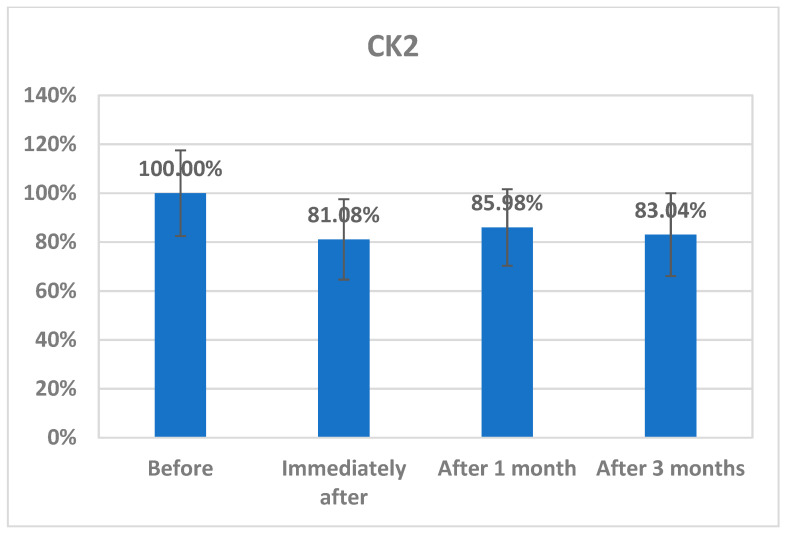
Percentage change in CK2 concentrations in plasma of autistic patients immediately, one month, and three months post AIT compared to before.

**Figure 2:**
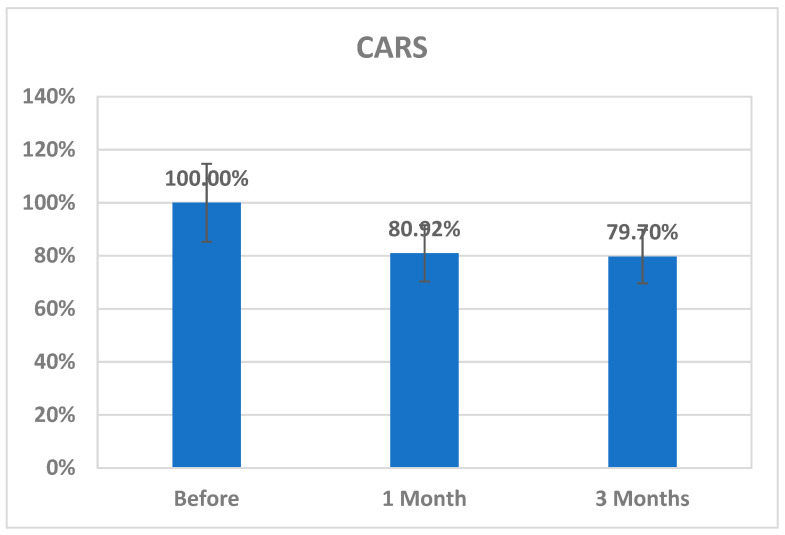
Percentage change in CARS scores of autistic patients one month and three months post AIT compared to before.

**Figure 3:**
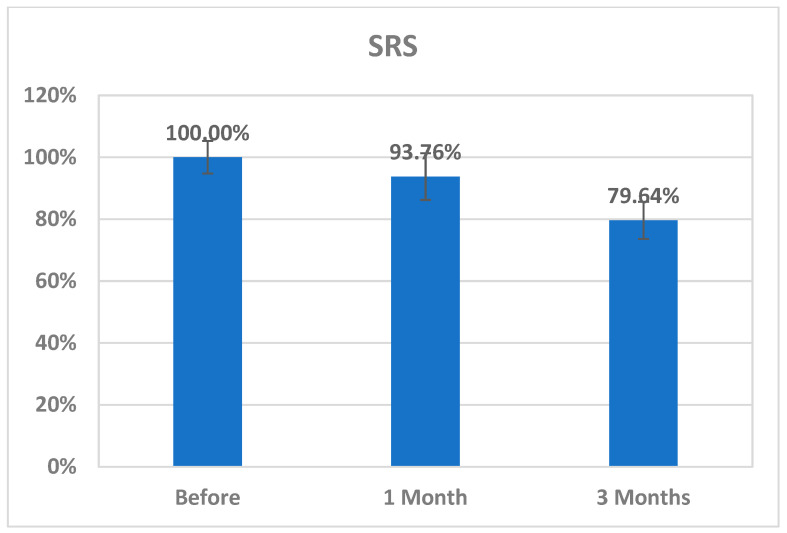
Percentage change in SRS of autistic patients one month and three months post AIT compared to before.

**Figure 4:**
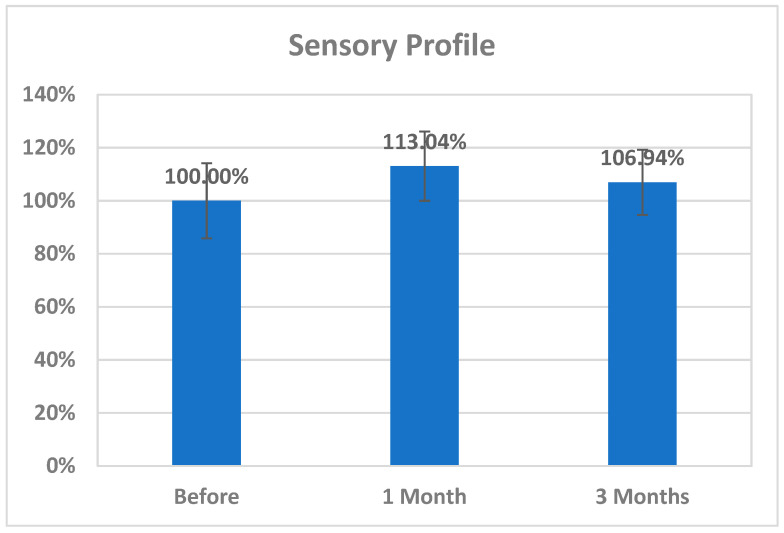
Percentage change in SP of autistic patients one month and three months post AIT compared to before.

**Figure 5:**
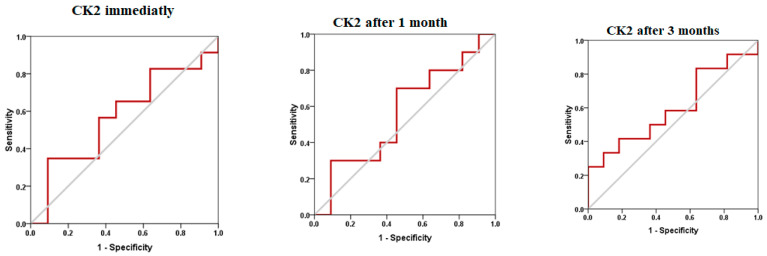

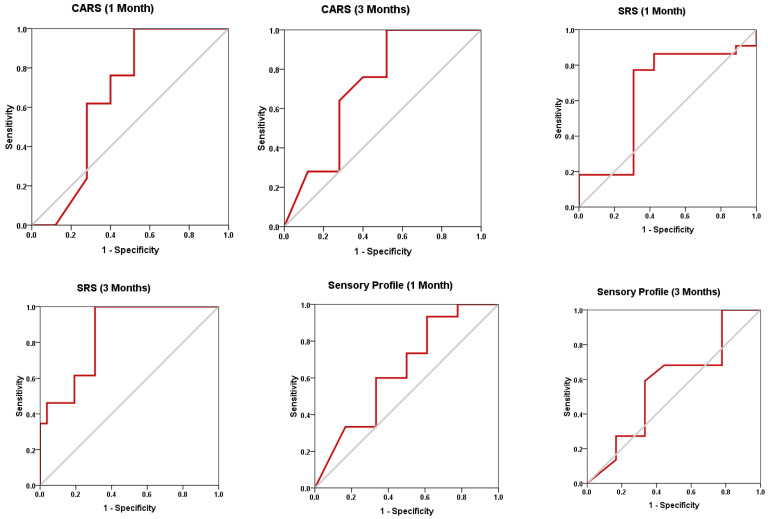
ROC curves of CK2, CARS, SRS, and SP post AIT intervention.

The changes in CK2 levels and the three behavioral rating scales (CARS, SRS, and SSP) before, immediately after, one month after, and three months after AIT are listed as means ± SD in Table 1, Table 2, Table 3 and Table 4. The plasma levels of CK2 significantly reduced by 18.92% immediately after AIT (*p* < 0.049), by 14.02 one month after AIT (*p* < 0.052), and by 16.96% three months after AIT (*p* < 0.046) compared to the results before AIT intervention (Table 1 and Figure 1). Scores of CARS, an indicator of autism severity, were decreased by 17% one month after AIT (*p* < 0.05) compared to before AIT (Table 2 and Figure 2). The total SRS scores significantly decreased (20.36%), and the total SSP scores were non-significantly increased three months after AIT (*p* < 0.612) (Table 3 and Figure 3). SP was non-significantly increase post AIT training (Table 4 and Figure 4). Table 5 demonstrates the remarkable decrease in GI symptoms among the AIT- treated participants. This suggests that for certain kids with ASD, AIT may exhibit a major therapeutic value. Table 6 and Figure 5 show the ROC analysis AUCs, specificity, and sensitivity of CK2 (immediately, one month after, and three months after), as well as SRS, CARS, and SSP (one and three months after). In the ROC analysis, the results can be interpreted as follows: AUC < 0.70, low diagnostic accuracy; AUC in the range of 0.70–0.90, moderate diagnostic accuracy; and AUC ≥ 0.90, high diagnostic accuracy. 

Table 2 describes paired samples *t*-test (parametric data) between each period (1 month and 3 months) and before using CARS scores.

Table 3 describes paired samples *t*-test (parametric data) between each period (1 month and 3 months) and before using SRS scores.

Table 4 describes paired samples *t*-test (parametric data) between each period (1 month and 3 months) and before using the sensory profile.

In recent years, complementary alternative medicine (CAM) treatments received increased attention from the scientific community: numerous studies have been conducted in order to examine the effectiveness and safety of CAMs in ASD. Unfortunately, there is a lack of evidence regarding the usefulness of CAM in ASD. There is remarkable contrast between the rate of use of CAM by families with autistic children and the lack of scientific outcomes of alternative treatments. One probable cause for this difference is that CAM remedies are generally considered as “natural”, with an optimum safety profile and fewer or the absence of side effects when compared to those of conventional drugs [40].

In the present study, AIT training intervention for three months significantly decreased CK2, with concomitant improvement of CARS, SRS, and SSP as measures of ASD severity, and GI as a co-morbidity of ASD.

Based on our current understanding of the etiology of ASD, many blood-based biomarker candidates have been investigated [41,42], particularly neurotransmitters [42], proinflammatory cytokines [42], markers of mitochondrial dysfunction [41,43], and markers of oxidative stress and impaired gut microbiota [44]. Most recently, Montanari et al. [45] reported that glutamatergic neurotransmission is highly indicated as an etiological mechanism in the pathophysiology of ASD, and it is considered to be directly related to ASD severity, identifying it as a potential target for novel management through which other etiological mechanisms could be also controlled or managed [41,45,46].

Proinflammatory cytokines (e.g., TNFα and IL-1β) negatively regulate glutamate transporter expression and activity, increasing extracellular glutamate concentrations [47]. In turn, immune activation increases cystine–glutamate exchanger (xCT) expression, possibly resulting in higher glutamate release and excitotoxic damage to oligodendrocytes [48,49].

In an attempt to determine the correlation between the reported significant decrease in CK2 (Table 1 and Figure 1) and the remarkable improvements in CARS and SRS scores, as measures of ASD severity previously related to glutamate excitotoxicity (Table 2, Table 3 and Table 4 and Figure 2, Figure 3 and Figure 4), it was of interest to emphasize the role of CK2 in phosphorylating glutamate receptors and/or transporters as protein components of the glutamate signaling pathway and to identify its involvement in pro-inflammation and apoptosis as critical events in ASD [50]. The significant improvement in CARS, SRS, and non-significant increase in SSP scored could be related to the significant reduction of CK2 activity one and three months post AIT. This could be explained on the basis that, in addition to CK2 apoptotic function, a number of studies have suggested its pro-inflammatory role and the possibility that CK2 pharmacological inhibition attenuates apoptosis and neuroinflammation as etiological mechanisms of many diseases, among which is ASD [51,52,53,54]. Moreover Canedo-Antelo et al. [55] recorded that CK2 inhibition rescues cultured oligodendrocytes from AMPA receptors and glutamate induced excitotoxic death. 

It is well accepted and proved that both peripheral and brain inflammatory responses are suggested to be associated with ASD-related behavioral symptoms. The suggested association between the AIT-induced CK2 downregulation and the observed improvement of SRS and CARS scores is supported by the work of Hafizi et al. [56], in which treatment with memantine and lenalidomide as anti-inflammatory drugs was associated with significant improvement in SRS and CARS scores. Moreover, the effectiveness of this treatment can be supported by considering the remarkable improvements in communication, daily living skills, social skills, and stereotypical behavior as measured by the Autistic Behavior Checklist (ABC) in response to treatment of inflammation using natural flavonoid luteolin. A positive correlation was recorded between behavioral improvement and the reduction in the serum levels of IL-6 and TNF following treatment with luteolin over a 12-month period [57,58]. The non-significant changes in SSP scores reported in the present study (Table 4 and Figure 4) could be attributed to the fact that their interpretation is complicated by limited content validity and considerable bias due to the multidimensionality of its integral parts. Williams et al. discouraged the use of the SSP total score and most subscale scores in children with ASD [59]. 

The altered gut flora in ASD has been linked to increased gut permeability, or ”leaky gut”, which allows bacterial metabolites to pass through the gut barrier and affect early childhood neurodevelopment in vulnerable individuals via the gut–brain axis. The studies of Raleigh et al. [60] provided fascinating preliminary insight into the intricate role of CK2 in the creation of TJs. Improvements in the transepithelial electrical resistance (TER) values, as well as a reduction in paracellular Na+-flux, resulted from treating the barrier function of Caco-2 cells with different CK2 inhibitors or the siRNA-mediated knockdown of CK2. Tight junctional occludin production increased when CK2 was suppressed, which might help to treat gut leakiness. Table 5 demonstrates the remarkable decrease in GI symptoms among the AIT-treated participants. Again, this could be related to the lower recorded levels of CK2 in AIT-treated patients. Lower CK2 levels might be accompanied by remarkable improvement in the tight junction integrity and intestinal permeability. 

ROC analysis presented in Table 6 and Figure 5 could help to suggest that among the four measured variables, while CK2 and SSP recorded low diagnostic value, with AUCs less than 0.7, CARS and SRS demonstrated moderate diagnostic value (AUCs of 0.7–0.9 range) as measures of the effect of AIT as CAM in ASD patients.

CK2 is considered as a potential therapeutic target due to its involvement in several neurological and psychiatric disorders. The vast range of inhibitors that are already accessible and might currently be in the hands of practitioners could be suggested as an intervention approach in ASD, in addition to AIT therapy [61].

**Scheme 2:**
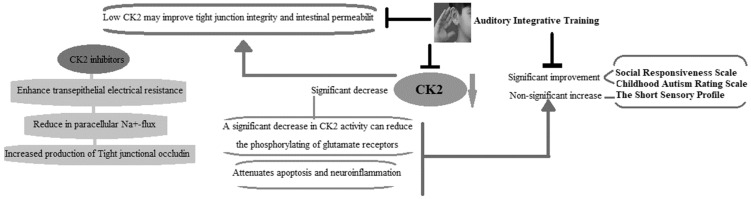
Illustrates how AIT affects scores for CK2, CARS, SRS, and SSP, in addition to GI as a co-morbidity of ASD.

Scheme 2 demonstrates the effect of AIT on CK2, CARS, SRS, and SSP scores, as well as GI as a co-morbidity of ASD. AIT significantly decreases CK2, yielding improved CARS, SRS, and SSP scores. A significant decrease in CK2 can improve CARS, SRS, and SSP scores in autistic patients through the reduced phosphorylation of glutamate receptors, diminished apoptosis, and decreased neuroinflammation. Tight junction integrity and intestinal permeability, which are required for a healthy gut, are improved by suppressing CK2 activity. The inhibition of CK2 can improve GI health in autistic patients via enhanced transepithelial electrical resistance and tight junctional occluding, with reduced paracellular Na^+^-flux. 

## 4. Conclusions

The findings reported here indicate the effectiveness of AIT as a tool of CAM as an intervention strategy in the treatment of ASD. Lower CK2 in AIT-treated participants, together with the significant improvement in SRS and CARS scores, indicate the possible ameliorative effects of neuroinflammation, apoptosis, excitotoxic, and leaky gut conditions as pathological mechanisms in autism. We propose that CK2 inhibition could be used as a promising target to lessen the severity of ASD.

## Figures and Tables

**Table 1:** Effect of auditory integrative training (AIT) on caseine kinase 2 (CK2) in children with autism (*n* = 25).

	Min.	Max.	Median	Percent Change	*p* Value
Before	0.406	5.317	2.447	100.00%	
Immediately after	0.462	5.476	1.984	81.08%	0.049
1 month after	0.533	4.36	2.104	85.98%	0.052
3 months after	0.127	5.851	2.032	83.04%	0.046

**Table 2:** Effect of auditory integrative training (AIT) on CARS scores of children with autism (*n* = 25).

CARS	Min.	Max.	Mean ± S.D.	Percent Change	*p* Value
Before	22	52	36.84 ± 10.95	100.00%	
1 month after	23	38	29.81 ± 6.24	80.92%	0.003
3 months after	22	37	29.36 ± 6.06	79.70%	0.008

**Table 3:** Effect of auditory integrative training (AIT) on SRS scores of children with autism (*n* = 25).

SRS	Min.	Max.	Mean ± S.D.	Percent Change	*p* Value
Before	138	203	179.42 ± 19.48	100.00%	
1 month after	78	218	168.23 ± 32.14	93.76%	0.002
3 months after	54	171	142.88 ± 27.23	79.64%	0.001

**Table 4:** Effect of auditory integrative training (AIT) on SP scores of children with autism (*n* = 25).

Sensory Profile	Min.	Max.	Mean ± S.D.	Percent Change	*p* Value
Before	87	190	144.56 ± 35.08	100.00%	
1 month after	129	190	163.40 ± 20.85	113.04%	0.223
3 months after	112	190	154.59 ± 23.25	106.94%	0.612

**Table 5:** Effect of auditory integrative training (AIT) on gastrointestinal (GI) symptoms before, one month, and three months post training.

GI Symptoms	Before	One Month	Three Months
Vomiting after eating	5	0	0
Reflux bloating	15	11	13
Diarrhea	8	1	1
Constipation	17	1	0
Colic	22	2	0
Itchy skin	12	1	1
Rash	6	0	0
Irritability	25	4	0

**Table 6:** ROC curves of CK2, CARS, SRS, and SP in different studied durations.

Duration	AUC	Cut-Off Value	Sensitivity %	Specificity %	*p* Value	95% CI
CK2 immediately after	0.573	2.076	56.5%	63.6%	0.401	0.402–0.745
CK2 1 month after	0.564	2.327	70.0%	54.5%	0.481	0.386–0.741
CK2 3 months after	0.598	1.893	50.0%	63.6%	0.253	0.433–0.764
SRS 1 month after	0.649	180.000	77.3%	69.2%	0.079	0.481–0.816
SRS 3 months after	0.848	176.000	100.0%	69.2%	0.000	0.742–0.954
CARS 1 month after	0.665	39.000	100.0%	48.0%	0.056	0.500–0.830
CARS 3 months after	0.717	38.500	100.0%	48.0%	0.009	0.570–0.864
Sensory Profile (1 month after)	0.643	145.000	93.3%	38.9%	0.164	0.453–0.832
Sensory Profile (3 months after)	0.577	156.500	59.1%	66.7%	0.407	0.391–0.763

## Data Availability

The datasets generated and analyzed during the current study are available from the corresponding author on reasonable request.
